# Bioimaging and Sensing Properties of Curcumin and Derivatives

**DOI:** 10.3390/ijms26104871

**Published:** 2025-05-19

**Authors:** Chiara Maria Antonietta Gangemi, Salvatore Mirabile, Maura Monforte, Anna Barattucci, Paola Maria Bonaccorsi

**Affiliations:** Department of Scienze Chimiche, Biologiche, Farmaceutiche ed Ambientali, Università degli Studi di Messina, V.le F. Stagno d’Alcontres 31, 98166 Messina, Italy; smirabile@unime.it (S.M.); maura.monforte@studenti.unime.it (M.M.); abarattucci@unime.it (A.B.)

**Keywords:** curcumin, luminescence, bioimaging, sensing, theragnostics, nano-formulation

## Abstract

Curcumin (Cur) is one of the most studied natural polyphenolic compounds, with many pharmacological properties and a luminescent skeleton. Natural fluorescent molecules are peculiar tools in nanomedicine for bioimaging and sensing, and this review focuses on the photophysical properties and applications of Cur in these biomedical fields. The first part of the review opens with a description of the Cur chemical skeleton and its connection with the luminescent nature of this molecule. The 1,6-heptadiene-3,5-dionyl chain causes the involvement of Cur in a keto–enol tautomerism, which influences its solvatochromism. The polyphenolic nature of its skeleton justifies the Cur generation of singlet oxygen and ROS upon photoexcitation, and this is responsible for the photophysical processes that may be related to the photodynamic therapy (PDT) effects of Cur. In the second part of the review, bioimaging based on Cur derivatives is reviewed, with a deeper attention paid to the molecular diagnostic and nano-formulations in which Cur is involved, either as a drug or a source of fluorescence. Theragnostics is an innovative idea in medicine based on the integration of diagnosis and therapy with nanotechnology. The combination of diagnostics and therapy provides optimal and targeted treatment of the disease from its early stages. Curcumin has been involved in a series of nano-formulations exploiting its pharmacological and photophysical characteristics and overcoming its strong lipophilicity using biocompatible nanomaterials. In the third part of the review, modifications of the Cur skeleton were employed to synthesize probes that change their color in response to specific stimuli as a consequence of the trapping of specific molecules. Finally, the methodologies of sensing biothiols, anions, and cations by Cur are described, and the common features of such luminescent probes reveal how each modification of the skeleton can deeply influence its natural luminescence.

## 1. Introduction

Curcumin (Cur) is a natural luminescent polyphenolic compound extracted from *Curcuma longa* L., used in traditional medicines, such as Ayurveda and Chinese, and known for its antioxidant and anti-inflammatory properties and antibacterial and antiviral activities [1]. It has also been extensively studied for its antitumor effects and applied to many types of cancer [2], including triple negative breast cancer, one of the most aggressive malignant neoplasms non-responding to conventional hormonal therapy [3]. Among compounds extracted from medicinal plants, Cur is one of the most studied [4]. For example, by restricting the research of articles to the last five years (2020–2024) in databases and search engines, more than 50,000 works appear with curcumin as the subject, highlighting that research in this field still represents a hot topic [5]. As a major active compound of turmeric, Cur is a yellow solid, usually sold in combination with demethoxycurcumin (17–20%) and bisdemethoxycurcumin (13–10%), but easily obtainable in its pure form by simple column chromatography. Cur is a lipophilic molecule, and its poor water solubility (10^−1^ μg/mL) greatly influences its bioavailability (~50 nM) in serum and its photophysical properties in water [6]. Although pharmacologically safe up to a dose of 8 g/day, Cur needs a high dosage to exert its therapeutic potential, possibly causing side effects regarding tolerance. For this reason, it is restricted to belonging to the Pan-Assay Interference Structures (PAINS) family [7]. Over the years, research has focused on the development of hydrophilic derivatives of Cur or nanotechnologies [8] that could bypass the critical issues of this polyphenolic compound and enhance its multivalent biological [9], chemical, and photochemical properties and/or deliver it in a specific biomedical targeted manner [4].

In this review, recent work on the use of curcumin in bioimaging and sensing is covered. The highly delocalized π-electron symmetric structure confers to Cur a natural luminescence. Its photophysical properties and fluorescence lifetime in solution have been summarized in the second section of the review. Curcumin-based fluorescent probes have some advantages compared to other natural and synthetic chromophores, which have been described in the third section of this review. However, they have some drawbacks, the main ones of which are poor water solubility and low chemical- and photo-stability for the labile β-diketone moiety. Nanoformulations, which are discussed in certain paragraphs, represent the most current answer to Cur problems as therapeutic and chromophore in diagnostic investigations. However, while the first aspect has been extensively covered in the literature [10,11], the application of Cur as a chromophore, which is the main subject of this work, has been much less addressed.

Derivatives in which the curcumin skeleton is maintained, but the sensitivity, solubility, and luminescence behavior are improved for use in bioimaging and sensing, will be part of this review, in which the efforts conducted by the authors in this field will also be highlighted.

## 2. Photophysical Properties of Curcumin

**Figure 1:**
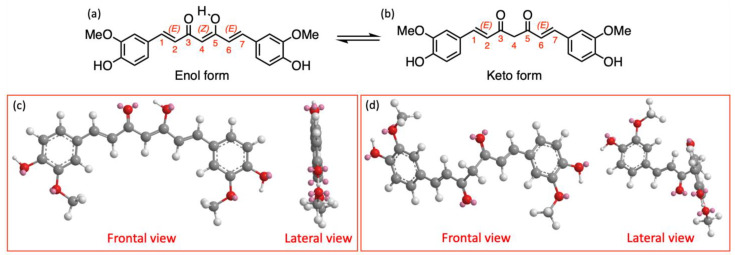
The enol (**a**) and keto (**b**) forms of curcumin. Minimized structures in frontal and lateral views of of enol form (**c**) and keto form (**d**).

In the early 2000s, there was the greatest number of scientific works on the chemical and physical properties of curcumin, which are summarized here, the majority of which were strictly related to its photophysical nature. Cur is a symmetric compound consisting of a seven-carbon skeleton, with an α,β-unsaturated β-diketone moiety, connecting two *o*-methoxy phenolic groups. Due to the presence of the 1,6-heptadiene-3,5-dionyl chain, Cur is involved in keto–enol tautomerism (Figure 1a,b), which is further enhanced by the presence of the diene moiety and *o*-methoxy phenolic groups, which influences not only the binding to nucleophiles, the hydrophobicity, and the polarity of the molecule but also its photophysical and biomedical properties [5].

Docking studies on structurally similar compounds demonstrate the importance of a correct tautomer in explaining the multivalent field of action of such natural drugs [12]. In general, the enol form of curcumin is more stable than the keto form for larger dipole moment of the enolic structure, which leads to the formation of strong intramolecular hydrogen bonds, keeping the structure with the two oxygens at the same side (Figure 1c). In contrast, the keto form has two C=O bonds in a “trans” orientation [13]. Nuclear Magnetic Resonance (NMR) studies, using different solvents at pH 3–9, have confirmed that (Z)-1,7-bis(4-hydroxy-3-methoxyphenyl)-5-hydroxy-1,4,6-heptatrien-3-one tautomer (structure (a) in Figure 1) is the only form present in solvents with a low dielectric constant. The keto form (structure (b) in Figure 1) can instead be detected only under certain special conditions, like low pH and protic solvents. Density Functional Theory (DFT) and X-ray diffraction studies in solid state indicate that the (Z)-enolic form gives extra stabilization to the molecule and allows conjugation between the π-electron systems of the two feruloyl moieties, while the keto tautomer is twisted [14] (lateral views (d) in Figure 1). Intramolecular hydrogen transfer barrier for the conversion of the keto form to the enol form of Cur has been calculated to be 4.78 kcal/mol.

Curcumin exhibits strong absorption in the UV/Vis region, with a maximum, due to S_0_–S_1_ electronic transition, in the range of 400 to 440 nm, while the emission is very susceptible to the solvent (460–560 nm), with a Stokes shift between 2000 and 6000 cm^−1^ [15]. The spectra in hexane and cyclohexane are structured, while a loss of the vibrational fine structure is observed moving to more polar solvents (e.g., DCM, MeOH). The presence of more than one shoulder probably indicates the presence of more than one isomeric form. The differences in the absorption spectra in different solvents are still not clear, but it seems they are correlated with the keto–enol equilibrium [16]. The keto form is less polar than the (Z)-enol form and may be stabilized in non-polar solvents, thus resulting in a blue shifted absorption spectrum. As the polarity of the solvent is increased, the keto–enol equilibrium is shifted towards the enol tautomer, thus resulting in a red shift of the band maximum and in the loss of the fine structure. Even at room temperature, the enol form is, in general, the predominant form in solution, confirming that the major photophysical properties of curcumin are dictated by this tautomer.

The fluorescence quantum yield, generally around 0.2 in polar solvents, is lower in cyclohexane and hexane and is significantly reduced in the presence of water due to predominant non-radiative decay processes. The fluorescence lifetime of the singlet state is very short (300–500 ps) and displays a multi-exponential decay profile. Fluorescence and triplet quantum yields are very low in all of the solvents due to either strong intramolecular or intermolecular hydrogen bonding. The phosphorescence spectra of Cur were recorded at 77 K in toluene and in ethanol in the wavelength region from 600 to 800 nm, with a broad maximum around 725 nm. The spectroscopic data for Cur recorded in different solvents have been conveniently summarized in various reviews [17].

**Figure 2:**
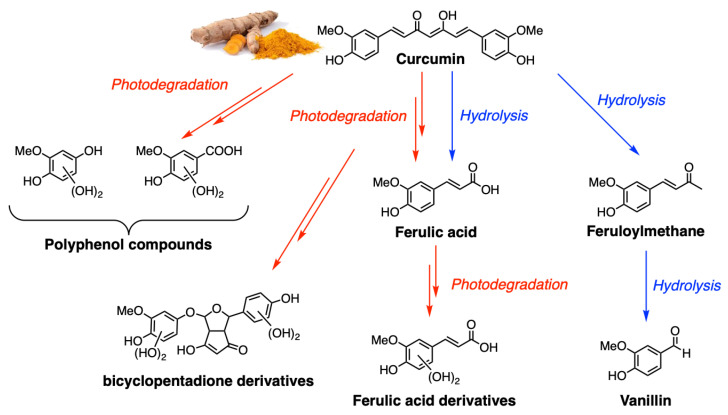
Products of “in-solution” and photodegradation of curcumin.

Curcumin undergoes faster degradation in solutions exposed to sunlight (Figure 2). The products identified during photodegradation are vanillin, ferulic acid, and other small phenols. A similar product distribution was obtained through chemical degradation [18]. This photodegradation involves the formation of the excited states. Upon photoexcitation, Cur generates singlet oxygen (^1^O_2_) and other ROS that are responsible for its photobiological and photodynamic activities. In such cases, the degradation of Cur after photoexcitation is supposed to proceed though the triplet excited state, which absorbs at 720 nm and reacts with oxygen to produce ^1^O_2_ [19]. Femtosecond fluorescence up-conversion spectroscopy has been used to reveal photophysical processes that may be related to the photodynamic therapy (PDT) effects of curcumin, including excited-state intramolecular hydrogen atom transfer (ESIHT) [20]. The results in MeOH and ethylene glycol show two decay components in the excited-state kinetics, a faster one of 12–20 ps and a slower one of about 100 ps. The fluorescence lifetime (FL) of curcumin undergoes biexponential decays in various solvents. The longer FL was recognized as a major component in the case of water that deviates from the rest of the solvents, while the shorter FL drastically changes from one solvent to another, without any correspondence with the polarity of the medium. A pronounced isotope effect has been shown by deuterated curcumin, suggesting that ESIHT is an important process on this time scale. The fast component in the excited-state kinetics is due to ultrafast solvation. Synchronous fluorescence spectroscopy was also used to investigate, in a fast way, solvent–Cur interaction and the specific solvent effects on the photophysical properties of the molecule.

## 3. Curcumin-Based Bioimaging and Sensing

Early diagnosis allows the identification of small tumors that have not yet spread to nearby organs, or viral or bacterial diseases, making, in many cases, the outcome of treatment better or even conclusive. Bioimaging and biosensing are the main techniques for an early diagnosis because of their ultra-high selectivity and sensitivity, and the research progress and application of Cur probes in these fields are relevant sections of this review.

Organic small-molecule dyes have shown a significant potential in diagnostic applications. Natural organic dyes such as the xanthene family, cyanine, coumarin [21], or curcumin have been deeply investigated, as they intrinsically possess some of the important properties for the design of diagnostic probes. Each class of these molecules offers different advantages, such as biocompatibility, tuneable photophysical properties, good fluorescence, and synthetic flexibility, but also have some drawbacks. For instance, cyanine is toxic and Cur is hydrophobic. However, the lipophilicity of Cur can overcome the cellular localization limits of other natural chromophores, such as hydrophilic rhodamine and coumarin. Due to its hydrophobic nature, Cur can localize within cell membranes, increasing their permeability and reducing thickness by protecting fatty acid residues from oxidation [22].

Many types of synthetic fluorescent materials have also been developed, such as gold nanoparticles [23], quantum dots [24], BODIPY derivatives [21], and so on, proving that fluorescent detection technology is a powerful investigating tool. Although these emerging materials are widely used as fluorescent probes, natural organic dyes like curcumin are not replaced because of their gratifying environmental compatibility and their cost-effectiveness. Furthermore, targeted modifications of their structural scaffold can turn them into *Near Infrared Region* (NIR) dyes. Small-molecule NIR dyes [25] are particularly appealing for their high photostability and strong fluorescence that does not interfere with natural protein fluorescence in in vivo imaging. Curcumin can be easily transformed into NIR-emitting dye by exploiting the chemical nature of β-diketone or by embedding it into silica nanoparticles, as described in the bioimaging and sensing sections.

### 3.1. Bioimaging

Bioimaging is a strong tool for exploring the internal functionality of the organism and its disorders; it can be used to aid illness management and therapy, as well as to detect, diagnose, and characterize problems in clinical settings [26]. Over the last two decades, emerging biological imaging technologies have generated tremendous biological discoveries. More efficient and accurate bioimaging results can be obtained by promoting interdisciplinary integration across material science, biology, medicine, and computational science, thus enabling early disease detection and treatment monitoring, improving therapeutic outcomes and reducing side effects. Good biocompatibility and high signal intensity must be balanced in the design of bioimaging materials to ensure the precise detection of cells within living organisms. The functionalization of these materials is equally important, allowing for specific targeted imaging through surface modification with targeting molecules. For the widespread use of bioimaging materials, high sensitivity must be maintained, minimizing toxicity and avoiding high production costs and complex syntheses.

**Figure 3:**
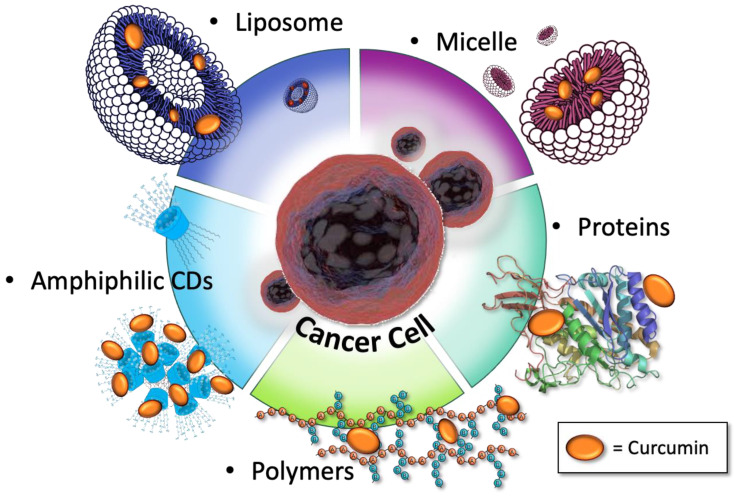
Nanocarriers hosting curcumin in their insides.

Cur exerts a persistent luminescence, biocompatibility, easy structural changeability, and a large spectrum of therapeutic effects so that, in several recent works, the theragnostic application of such molecules has been proposed. To bypass its critical issues and highlight its use in nanomedicine, most of the papers, some of which are here reviewed, describe different types of luminescent curcumin nano-formulations, along with their major advantages [27]. Nanomedicine is a broad term that includes all the nanotechnologies that have been developed since the 1960s. In this section of the review, we want to highlight two of the fields in which nanomedicine is involved—molecular diagnostic and nano-pharmaceuticals [28]—in both of which small molecules, such as Cur, are used to increase efficiency and reduce the harmfulness of diagnostic and therapeutic methods. Recently, both the possible mechanism and the therapeutic effect of Cur nanopreparations on gastrointestinal disease, a major health problem, have been reviewed [29]. Nanoformulations have been developed either to achieve a systemic increase in curcumin biodistribution or to accomplish, by specific targeting, a higher concentration of Cur in specific cells, tissues, or organelles. In the nanoscale dimension (usually less than 100 nm), the molecules can exhibit unique optical, electrical, and mechanical properties, resulting in advances in imaging, sensing, and drug delivery. As described in the following subsections, Cur was embedded into a hyperbranched and hydrophilic polymeric matrix, into liposomes structures using amphiphiles molecules, loaded into mesoporous or biocompatible materials, or into inclusion complexes (e.g., cyclodextrins), as shown in Figure 3. These nanoformulations improve Cur bioavailability under physiological conditions and avoid photodegradation for light exposure. Nanocarriers like amphiphilic cyclodextrins (αCD) [30], liposomes, and polymeric micelles can also help to overcome the mono-therapeutic complications in cancer treatment protocols, hosting in their insides more than one active principle. The combination of Cur with a fluorescent nanomaterial, such as carbon dots (CDs), can also enrich the luminescence characteristics of both components and yield luminescent material with broader emission spectra, which is very useful in bioimaging applications. Furthermore, because the poor solubility of Cur is responsible for the observed instability in water, its chelation with metal ions (M^2+^) or with BF_2_ moiety improves stability and lifetime in aqueous media. Although preclinical experiments have shown that nanoformulations can improve Cur efficacy, studies are still insufficient for in vivo applications.

The most explored medical area of nanomedicine lies in tumor therapy [31], but other fields of clinical medicine, such as infectious diseases [32] that are responsible for the most serious public health problems, are now benefiting from it.

#### 3.1.1. Polymeric Curcumin-Based Nanoformulations

**Figure 4:**
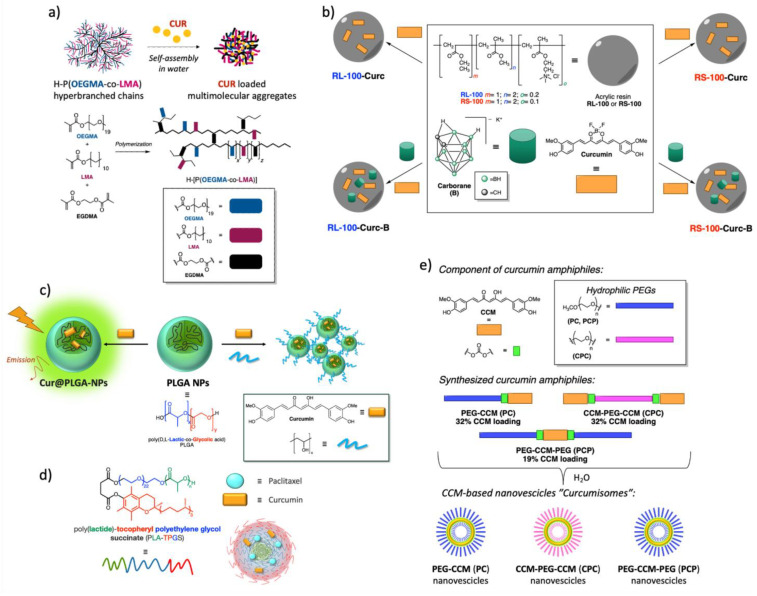
Coating of curcumin with acrylic resins. (**a**) Cur-NPs composed of Pluronic F127 and H-[P(OEGMA-co—LMA; (**b**) Cur-NPs with carborane coated on RL-100 and RS-100 resins; (**c**) Cur-PLGA- NPs; (**d**) Cur and paclitaxel PLA-TPGS micelles; (**e**) Cur-polyethylene glycol based “curcumisome”.

The coating of drugs with acrylic resin is widespread in the pharmaceutical field and is used to increase solubility, absorption, and bioavailability and reduce the damaging effects of drugs on the body. Therefore, various Cur-based nanoparticles (NPs) use the coating principle [33], as shown in Figure 4. Nanocarriers based on mixed copolymers composed of Pluronic F127 and hyperbranched poly[(ethylene glycol) methyl ether methacrylate-co-lauryl methacrylate] (H-[P(OEGMA-co—LMA)] were prepared using, as the best methodology, the co-solvent approach. The copolymers were combined to generate micelle-like nanostructures with small average radii. Curcumin loading of such systems produced very stable and fluorescent novel cur-nanomaterials that were studied in fetal bovine serum to simulate bloodstream conditions, exhibiting outstanding stability and appearing as promising candidates for bioimaging applications (Figure 4a) [34].

Curcumin and carborane were coated with acrylic resin RL-100 and RS-100 into spherical NPs using the oil-in water strategy. Four different types of Cur fluorescent complexes were prepared and characterized by infrared spectroscopy. Their affinity and biocompatibility were studied by the imaging of HeLa cell lines under a laser confocal microscope. They all enhanced the compatibility between curcumin and tumor cells of the solubility and bioavailability of the natural anticancer drug (Figure 4b) [35].

Nanoparticles of poly (D,L-lactide-co-glycolide) acid (PLGA) loaded with Cur and embedded in a polyvinylalcohol (Cur@PLGA-NPs) were prepared and examined for their luminescent and Cur-carrier properties. Cur@PLGA-NPs exhibited high fluorescence intensity due to aggregation-induced emission (AIE) and enhanced apoptotic effects on CT26 cells with respect to free Cur, suggesting that the amorphous AIE formulation can be promising for its imaging and bioactive capabilities (Figure 4c) [36].

The encapsulation of Cur into poly(D,L-lactic-co-glycolic acid) led to luminescent NPs with an enhanced biocompatibility and solubility compared to free Cur. The polymeric encapsulation did not cause significant changes in the luminescent properties of Cur, and particles exhibited good uptake in human cancer cells, showing promising properties as probes for bioimaging and, in addition, as antibacterial agents (Figure 4c) [37].

Cur and paclitaxel were loaded onto poly(lactide)-tocopheryl polyethylene glycol succinate-based micelles (Figure 4d). Two types of curcumin nanoparticles were obtained and characterized. The fluorescent properties of Cur were maintained. The results showed that these NPs exhibited better uptake to MCF7 monolayer and MCF7 spheroid cells than that of pure Cur. Both these observations provide evidence for the potential of such NPs for cancer theragnostics [38].

Cur was derivatized with hydrophilic poly(ethylene glycol), and the self-assembly of amphiphilic Cur-polyethylene glycol conjugates produced novel biodegradable nanovesicles that the authors called “curcumisome” (Figure 4e). They showed high water dispersibility and efficient internalization into human cancer cells, with strong fluorescence maintained for a prolonged period, while a small amount of such nanovesicles was detected in normal cells, producing only a weak fluorescence. The mechanism of internalization was unclear, but these nanovesicles induced apoptosis of cancer cells, with promising properties of the theragnostic biomaterial [39].

#### 3.1.2. Carbon Dot Curcumin-Based Nanoformulations

**Figure 5:**
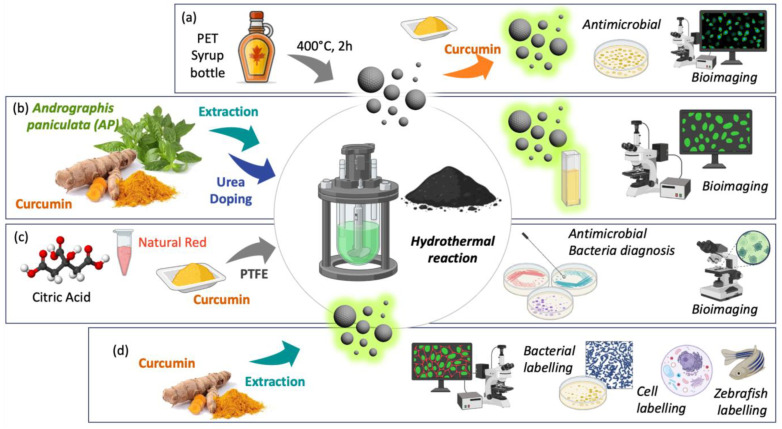
Nano-formulations of curcumin based on carbon dots. (**a**) Cur-CD from PET syrup bottle; (**b**) *Andrographis paniculata* and curcumin CDs; (**c**) Citric acid-Cur-natural red CDs; (**d**) Cur-CDs from turmeric extract.

Carbon dots (CDs) are carbon-based nanomaterials used in a wide range of applications as novel drug delivery systems for their high biocompatibility, easy synthesis, excellent chemical inertness, and low toxicity. A recent environmentally friendly and sustainable approach proposes discarded syrup bottles as a carbon source for CDs, which were combined with Cur to give a novel luminescent hybrid material for applications in bioimaging and antibacterial treatments. This combination overcomes the limitations of curcumin and enhances its antimicrobial properties. In association with the luminescence of CDs, it provides a contribution to the integration of sustainable nanotechnologies with bioactive natural materials (Figure 5a) [40].

The nano-structurization of chlorophyll from *Andrographis paniculata* and curcumin into CDs, followed by nitrogen functionalization, led to the formation of CD particles with good fluorescence characteristics and biocompatibility. The urea functionalization produced nanostructures with tunable optical properties and enhanced solubility of the nano-formulation, which could penetrate cells with an optimal dose for bioimaging assays (Figure 5b) [41].

Spherical Cur quantum CDs were synthesized using a hydrothermal method, with a fluorescence absorption in aqueous solution at 555–850 nm and a maximum emission at 635 nm. The Cur-based nanomaterial has excellent storage, light stability, and good cell compatibility and can be used as a biological imaging agent. They also showed an improvement in efficiency of ROS production compared to free Cur. Under the irradiation of a Xenon lamp, the Cur carbon quantum dots inactivated Gram-positive and Gram-negative bacteria, destroying the integrity of the cell membrane, thus indicating that they are a candidate material for antimicrobial photodynamic therapy (a-PDT) (Figure 5c) [42].

CDs from Cur were prepared by hydrothermal synthesis, and they exhibited excellent fluorescence intensity and high photostability. Their biolabeling properties were tested on Gram-positive and Gram-negative bacteria and on some cancer cell lines, resulting in low cytotoxicity and good enlighten potential. Zebrafish embryos were imaged and in vivo toxicity was evaluated, confirming the biolabeling potential of such nanoparticles (Figure 5d) [43].

#### 3.1.3. Curcumin-Based Inorganic Nanoformulations

Curcumin was used as a modifying agent on the surface of copper sulfide (CuS) colloidal nanoparticles that were transformed, in a three-step process, into CuS nanosheets through sulfidation. These nanosheets exhibited fluorescence and surface plasmon resonance within the near-infrared (NIR) spectrum, thus showing good potential for bioimaging. After sulfidation, the cellular toxicity in HeLa cells was reduced by several orders in respect to CuNPs before this process. Thus, this new chalcogen material appears interesting for future imaging and sensing applications [44].

Graphene oxide was functionalized with a Cur derivative via polycondensation reactions to obtain a composite that was fully characterized and showed good dispersion in phosphate buffer and significant photoluminescence. In vitro and in vivo bioimaging results confirmed its low cytotoxicity, good photostability, and bright bioimaging [45].

Cisplatin and Cur, co-incorporating hybrid CaCO_3_ nanoparticles, were used to prepare a multichannel Ca^2+^ nanomodulator by a facile one-pot strategy with in situ synthesized polydopamine (PDA) as a template, involving a gas diffusion procedure. Further modifications, such as PEGylation, of the nanomodulator allowed its selective accumulation in tumor tissue. The nanomodulator enters the tumor cells and induces multilevel mitochondrial damage. The fluorescence imaging of Cur, combined with the photoacoustic imaging of PDA, facilitate the visualization of the nanomodulator, depicting it as an example of multimodal bioimaging-guided organelle-targeted cancer therapy [46].

Gold nanoclusters with a particle size of 1–3 nm, conjugated with Cur (Cur-AuNCs), were synthesized using a green procedure. The luminescence of Cur was maintained within the cluster, suggesting the advantage of using the natural polyphenol for bioimaging. HeLa cells were treated with Cur-AuNCs that showed significant cytotoxicity and morphological damages against these cervical cancer lines, while showing a very negligible toxicity against COS-7 normal kidney cells. The results demonstrated that Cur-AuNCs can be studied for anticancer therapy and bioimaging applications [47].

#### 3.1.4. Curcumin-Based Liposomes

Liposomes are extensively used in drug delivery for their good biocompatibility and bioavailability and high adaptability for loading either hydrophilic drugs, which occupy the water phase, or hydrophobic drugs, located in the lipid membrane. The decoration of liposomes with active targeting ligands can precisely direct the drug in the required body location. For instance, dimyristoyl phosphatidylcholine (DMPC), phosphatidylethanolamine (PE), and cholesterol were used to prepare liposomes that were loaded with Cur and carbon dots. Anti_CD44 antibodies were conjugated on the liposomal surface to give a targeting nanocarrier. After the characterization of the vesicles, in vitro analysis was conducted to investigate the theragnostic properties of such liposomes. They showed an enhanced effect on cancer cells in respect to Cur-loaded liposomes. Their bioimaging potential was studied on U-87MG and HaCat cell lines, which suggested that these nano-formulations can be a useful tool for real-time diagnostic [48].

Chemiluminescent peroxyoxalate reaction was used to prepare liposomes, composed of bis-(2,4,6-trichlorophenyl) oxalate and Cur as fluorophore, that can detect H_2_O_2_ and serve as therapeutic agents with antioxidant and anti-apoptotic activity. Cur acts either as an oxalate activator or a photosensitizer, causing cell damage [49].

#### 3.1.5. Boron Difluoride Curcumin-Based Nanoformulations

**Figure 6:**
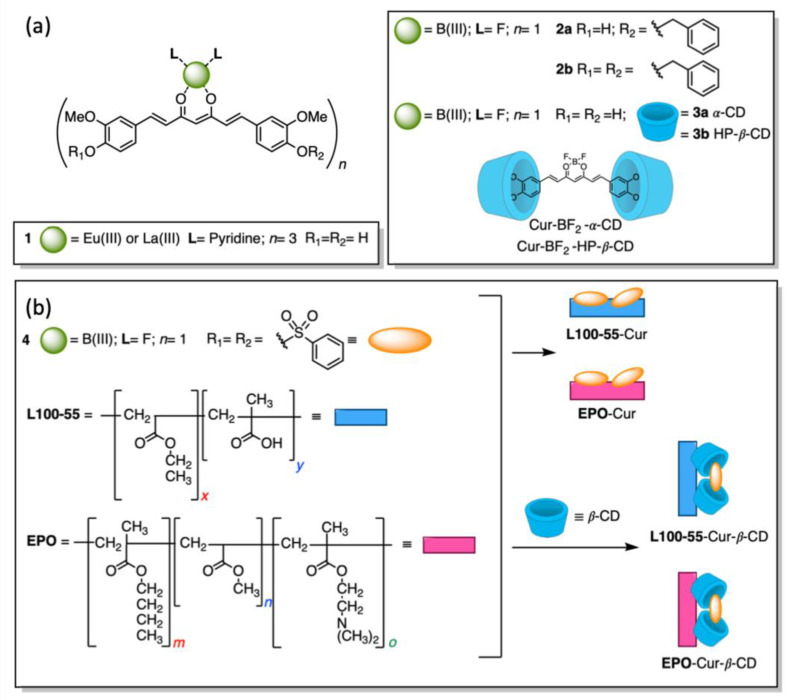
Metal–curcumin complexes and their applications in bioimaging. (**a**) ML_2_-Cur and its inclusion complexes with α-CDs; (**b**) ML_2_-Cur on acrylic resins and ML_2_-Cur β-CDs inclusion complexes on acrylic resins.

Cur is part of the large family of acac (acetylacetonate) ligands, whose b-diketone chelating group is widely used in coordination chemistry due to the stability of its complexes with a variety of metal ions. This stability originates from Cur chemical properties arising from the keto–enol tautomerism (already illustrated in paragraph 2 and Figure 1), which provides the binding in different coordination modes to the two oxygen atoms [50]. For instance, Eu(III) and La(III) complexes (compound **1** in Figure 6a) with Cur and Pyridine were synthesized and fully characterized. In the novel complexes, the oxygen atoms of Cur and the nitrogen atoms of pyridine were coordinated to the rare earth ions. The two complexes exhibited good luminescence by two-photon microscopy in the NIR region and in highly polar solvents. Moreover, a strong emission was observed for MCF-7 cells labelled with the Eu(III) complex, indicating that the two rare earth complexes can be used for diagnostic applications [51].

The incorporation of BF_2_ into a chromophore unit usually allows the boosting of some of the photophysical properties of the fluorescent dye, such as absorption coefficient and quantum yield, Stokes shifts, and tunable absorption/emission profiles. Boron dipyrromethene (BODIPY) derivatives, for instance, have attracted wide research interest and found applications in a variety of fields. In this context, the incorporation of BF_2_ into the 1,3-diketone moiety of Cur skeleton shifts its absorption out of the UV region, with a maximum between 475 and 503 nm, increases its photostability, reduces nonradioactive relaxation rates, and, in some cases, enhances the reactivity toward the Knoevenagel reaction. Indeed, the fluorescence emission spectra of BF_2_-Cur are red-shifted, allowing more biological applications. For this reason, a considerable number of works have been published on BF_2_-modified Cur derivatives, and some of the results have been illustrated in papers and reviews [52,53,54].

Two benzylated curcumin derivatives (Figure 6a) were synthesized to be used as fluorescent probes. The synthesis of compound **2** involved the substitution of the phenolic group with an O-benzyl group as a weak donor and the inclusion of BF_2_ as an acceptor. Both molecules (compounds **2a** and **2b**) exerted a minimal cytotoxicity against cancer cell lines and selectively visualized the cytoplasm of SVG (non-tumoral) and U-251 (tumoral) cell lines or the nucleus when confocal fluorescent microscopy (CFM) was used at 405 up to 560 nm. The potentiality of such curcumin-based probes relies on the great permeation of cell membranes that allows their use in live-cell imaging and biomedical studies [55].

Two inclusion complexes of α-cyclodextrin (compound **3a**) and hydroxypropyl β-cyclodextrin (compound **3b**) with Cur, modified with boron trifluoride ether (Figure 6a), showed excellent photophysical properties and an emission maximum in the range of 550–565 nm. Both complexes had certain inhibitory effects on the proliferation of cancer cells and provided good results in the fluorescence imaging of HCT-116 and Hela cells [56].

The BF_2_-Cur sulfurated derivative **4** in Figure 6b was coated, alone or in the inclusion complex with a β-cyclodextrin, in two types of acrylic resin (L-100-55 and EPO) using the oil-in-water strategy. Four different types of fluorescent complexes were prepared and characterized (Figure 6b). Transmission electron microscopy demonstrated that the acrylic resin coats Cur into rods and clusters. The fluorescence imaging of HeLa cells showed a good biocompatibility of such complexes. Their emission intensity was significant [57].

#### 3.1.6. Mesoporous Curcumin-Based Nanoformulations

**Figure 7:**
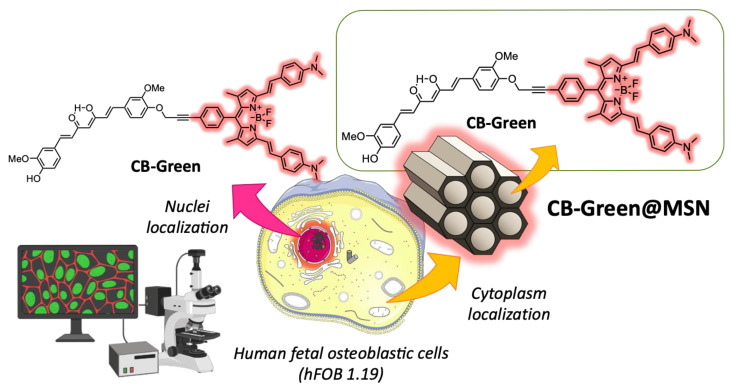
Bichromophoric system CB-green in MCM-41 mesoporous nanoparticles.

Recently, mesoporous materials have gained tremendous attention in several significant applications, from sensing, energy conversion and storage, and catalysis to biomedicine, for the unique features that their various nanostructures exert, including good biocompatibility, high surface areas, large pore sizes (2–50 nm) suitable for drug delivery, tunable pore structures, controllable framework compositions, high stabilities, and easy functionalization [58]. Among mesoporous materials, mesoporous silica nanoparticles (MSNs) have been extensively utilized for biomedical purposes [59]. MCM-41 NPs, with an average size between 50 and 80 nm and meso-structured pores with 2D hexagonal arrangement, exhibit singular features, such as ordered pore networks, large pore volumes (0.6–1 mL/g) with a very narrow and tunable pore size distribution (2–10 nm), and high surface areas (600–1000 m^2^/g) [60]. Furthermore, MCM-41 NPs show biocompatibility and inertness and are able to deliver organic molecules, even in aqueous-based formulations, avoiding toxic organic solvents. They were chosen as recipients of a Cur-based luminescent probe, that images in the NIR region, to limit the drawbacks of its high lipophilicity, such as aggregation and shortening of emission time. The NIR fluorophore was conceived as a bichromophoric system, as shown in Figure 7, with Cur linked to a BODIPY derivatives. This approach overcomes the inability of Cur to image the NIR region, because of the short absorption wavelengths, poor chemical and photochemical stability, and brightness loosing upon photo-illumination. The bichromophoric system is characterized by efficient photoinduced energy transfer, active within the bichromophoric species, that allows for a high difference between the excitation and the emission energy, thus suppressing photon scattering and diminishing tissue autofluorescence. It also allows the biological material (human fetal osteoblastic cell line, hFOB 1.19, and human bone osteosarcoma epithelial cells, U-2 OS) to be illuminated in the NIR by exciting it in the blue. In addition, it targets the nucleus of the studied cell lines, thus supporting the idea that small-size dye molecules are desirable tools in bioimaging since they quickly enter the cell, locate the target region, and, in this specific case, do not induce any stress to cells for up to 5 days. The encapsulation of Cur-BODIPY-based dyad inside MCM-41 silica nanoparticles allows them to mask Cur’s lipophilic nature, overpass the cellular membrane, and locate within the cytoplasm without losing brightness. The bichromophoric system maintains a very efficient energy transfer, even within the hybrid silica system, which confirms the viability of the encapsulation strategy for imaging purposes [61].

**Figure 8:**
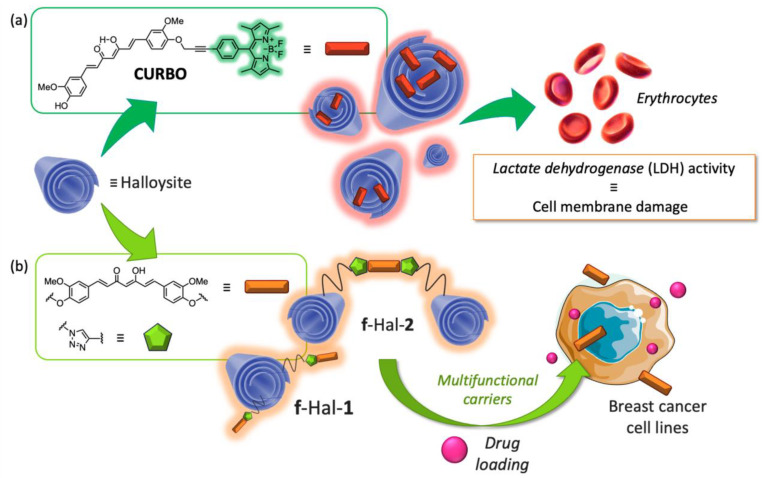
(**a**) Bichromophoric system CURBO in halloysite mesoporous NPs; (**b**) Curcumin-halloysite nanotubes system.

In continuing the exploitation of new nano formulations for application in bioimaging and theragnostics, a new Cur-BODIPY bichromophoric system, shown in Figure 8a, was loaded into halloysite nanotubes (HNTs) [62]. Halloysite is a mesoporous material, and particularly, a natural aluminosilicate clay from the kaolin group, with a predominantly hollow tubular structure. Generally, the inner lumen, mostly composed of aluminol groups, is in the ranges of 10–30 nm, while the outer diameters of the tubes are 40–70 nm. HNTs possess positive charges in the inner lumen and negative ones in the external one, which consists of silicon dioxide. Due to the different chemical composition, halloysite nanotubes can be selectively functionalized at the inner and/or outer surfaces, leading to the synthesis of several interesting nanomaterials with promising biological activities. Halloysite biological safety has been reported by both in vitro and in vivo studies. In general, the HNTs can penetrate the cellular membrane surrounding the cell nuclei, and modification of the tube surfaces also allows them to penetrate the nucleus membrane. So far, several biologically active molecules have been simultaneously loaded onto HNTs, obtaining synergistic cytotoxic effects (Figure 8b) [63]. The Cur-BODIPY dyad is characterized by an intense fluorescence band in organic solvent that reveals a photoinduced energy transfer, active within the bichromophoric species. Its biocompatibility and photophysical behavior were studied preliminary using ASC52Telo, h-TERT immortalized adipose-derived mesenchymal stem cells. It was found to be not cytotoxic up to 10 μM and lights up in green ASC52Telo cells. However, its photophysical behavior in aqueous media was complicated by the effect of stochastic aggregation caused by the poor solubility. The HNT/dyad nanocomposite showed a significantly increased biocompatibility with respect to the dyad alone, and an increased aqueous solubility that leads to a spectral widening of its emission up to the red region (NIR). The nanocomposite had a high propensity to cross cell membranes, resulting in a massive cell uptake. It lit up ASC52Telo cells with both intense green and red fluorescence, while it was excited in the blue portion of the absorption spectrum, showing properties of a dual-channel emissions probe. This type of dye is particularly fascinating since it can minimize the autofluorescence detection of the cell medium and allow interesting microscopy applications. Moreover, this Cur- BODIPY bichromophoric system was used as a model to study the transport mechanisms of HNTs. The results have shown that the internalization process of nanotubes occurs in an energy-dependent manner and that the dyad is localized in the cytoplasmic area of some cell lines, particularly in the perinuclear region [64].

### 3.2. Sensing

Due to the inherent luminescence and the highly symmetric structure of π-delocalized electrons, with an α,β-unsaturated-b-diketone moiety, Cur has been widely employed in optical and electrochemical sensors because it can also boost optical properties when its skeleton interacts with nanostructures [62] or metal ions and is capable of enhancing the electrochemical signals, with the central methylene group having a crucial role in its redox behavior. There is a third important reason for the widespread use of Cur-based sensors: Cur is an environmentally friendly and low-cost molecule that can be easily biodegraded on disposal and possesses a broad range of pharmacological and biological activities. In this section, we addressed our attention to optical sensors, in which Cur acts as a fluorophore, as well as its recognized pharmacological properties. We did not report on the electrochemical sensing of Cur, for which recent reviews are available [65].

#### 3.2.1. Curcumin-Based Sensing in Food and Environment

Environmental pollution and food quality and preservation are two major global issues in the world and hot topics for Cur-based sensing. Several significant reviews have been, even recently, published on both issues [66,67,68]. Fluorescence and imaging techniques, utilizing the color change of luminescence to achieve the imaging of targets, is highly sensitive, simple, and has low cost, and for these reasons it has attracted attention for food analysis [69]. For instance, a ratiometric luminescent sensor was designed and synthesized for the detection of salicylic acid (SA) in rice. SA is considered an important phytohormone that contributes to increasing the resistance of plants against biotic and anti-biotic stresses. On the other hand, the abuse of SA and its derivatives can cause serious allergic reaction of angioedema and asthma. The probe was developed with the assembly of CDs, with silk as the raw material and Cur as luminescent and potential captor of SA and Fe^2+^ ions. Specifically, the Fe^2+^ ions were employed to capture, on one side, the Cur α,β-unsaturated-diketone moiety, and on the other side, the SA by a ternary complex, avoiding the interference of other metal ions. The response to the formation of the ternary complex was an increase in the emission of the “turn-on” sensor. The good recovery of SA in rice confirmed the potentiality of such a sensor [70].

#### 3.2.2. Curcumin-Based Sensing in Neurodegenerative Diseases

Alzheimer’s disease (AD) is one of the most common neurological syndromes, distinguished by a progressive decline in cognitive functions such as memory, language, and executive skills, independent of normal aging-related cognitive changes. The pathology of AD is characterized by a synergistic interplay between neurofibrillary tangles, composed of hyperphosphorylated tau proteins, and β-amyloid plaques in the brain. The accumulation of these protein deposits has been associated with widespread neuronal damage and atrophy. Hyperphosphorylation and the dissociation of tau proteins produce oligomers that ultimately form neurofibrillary tangles. Amyloid plaques arise from the proteolytic cleavage of amyloid precursor proteins that can undergo brain internalization, assume a β-folded or β-pleated configuration, and subsequently accumulate into extended fibrils and aggregates, constituting plaques, formed through free radical activity [71].

**Figure 9:**
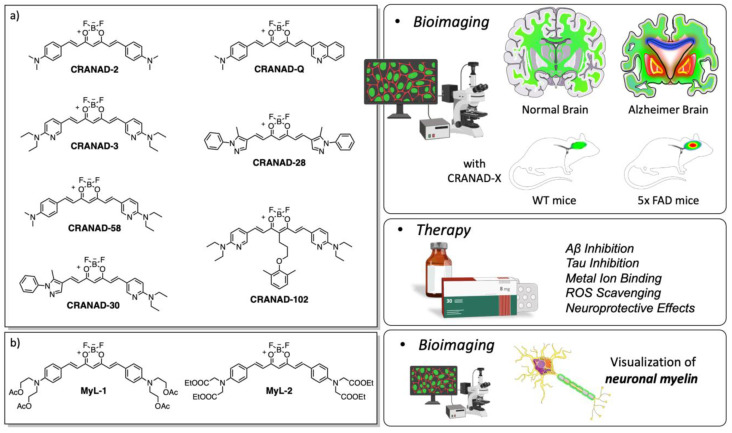
Luminescent AD sensors of CRANAD family. (**a**) CRANAD, (**b**) MyL-1-2.

Research has shown the neuroprotective effects of Cur in AD through various mechanisms. It has been observed that Cur promotes tau protein inhibition and β-amyloid plaque disaggregation, regulates the process of oxidation, and induces anti-inflammatory mediators [72]. It felt therefore natural to involve Cur in the synthesis of AD sensors. For instance, the CRANAD (which stands for the initial and last name of the first author, C. Ran, as well as for Alzheimer’s disease, AD) [73] family of small molecules, with BF_2_-Cur, well describes the evolution in the potentiality of such AD sensors, as depicted by several reviews on this argument and in Figure 9a [54,74].

Recently, a small library of compounds, possessing structural features of the CRANAD family but called by paper authors **MyL-1**-**2** (Figure 9b), have been projected for ex vivo myelinic targeting. One of these molecules, **MyL-1**, shown in Figure 9b, enables the direct visualization of sphingomyelin in the brain with a strong fluorescence, high specificity, and excellent photostability. The authors emphasize its role in studying and understanding the intricate process of myelinogenesis [75].

**Figure 10:**
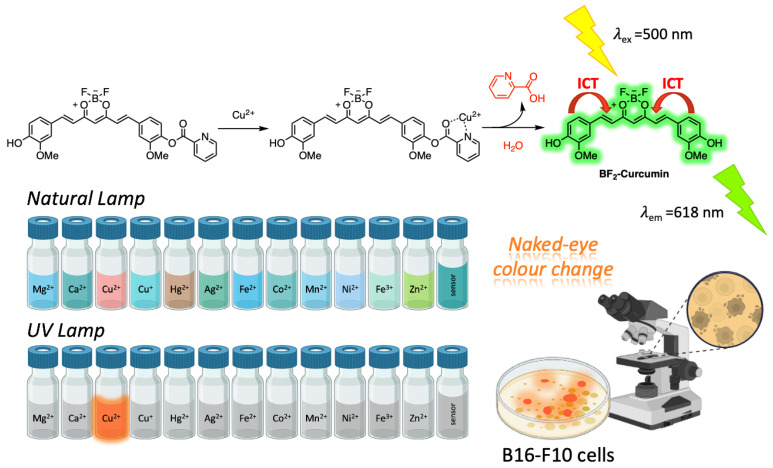
Cu^2+^ sensing mechanism of 2-picolinyl BF_2_-Cur complex.

Copper is the third most abundant essential element in biological systems, where it plays a vital role in various physiological processes. Either a deficiency or high levels of this ion can cause serious problems for human health, such as neurodegenerative (e.g., Alzheimer’s, Parkinson’s, and Wilson’s disease) or cardiovascular diseases. Because of the effects of copper ions on human health, sensing these ions has become essential [76]. Some examples of Cur-based luminescent sensors for the detection of Cu^2+^ have been reported. One of these was synthesized by a one-step reaction. The results demonstrated a high selectivity towards Cu^2+^, in phosphate buffer solution, over other metal ions. The excellent imaging of Cu^2+^ in living MCF-7cells was also evaluated by laser confocal fluorescence microscopy. The addition of Cu^2+^ enhanced the fluorescence intensity in cells, and the Cur-Cu^2+^ sensor exhibited better cell imaging than the sensor alone. Further localization experiments revealed that the Cur-Cu^2+^ sensor was mainly distributed in the cytomembrane [77]. Exploiting the luminescence properties of the BF_2_-Cur complex, a sensor was synthesized where the picolinic acid was linked to the BF_2_-Cur complex, based on the well-known characteristic of Cu^2+^ of inducing the hydrolysis of picolinate in aqueous solution (Figure 10). As a matter of fact, the sensing mechanism was discussed as follows: The intramolecular charge transfer of BF_2_-Cur complex was weakened by the ester bond with 2-picolinic acid. Once the picolinate was hydrolysed under the action of Cu^2+^, the strong emission intensity of the BF_2_-Cur complex was recovered. The sensor exhibited naked-eye color change in the presence of Cu^2+^ from blue to pink in a buffer solution. Moreover, imaging experiments were conducted for B16-F10 cells to demonstrate the potential of the sensor in biological systems [78].

#### 3.2.3. Curcumin-Based Sensing of Biothiols

The accurate sensing and tracing of biothiols cysteine (Cys), homocysteine (Hcy), and glutathione (GSH), the most abundant small molecules in cells, are of great significance for understanding their physiological functions and the related issues. Cys is a precursor of GSH, acetyl coenzyme A, and sulfur-containing proteins in organisms. It affects the oxidative stress of cells and participates in the regulation of the body’s intestinal function, lipid metabolism, and other processes. Usually, the abnormal levels of Cys have a close relationship with many diseases like neurotoxicity, hair depigmentation, oedema, liver damage, and skin lesions. Hcy has great significance in disease diagnosis and treatment, especially as a key indicator of ischemia–reperfusion injury of the heart. The total concentrations of Hcy in plasma are also related to birth defects, cognitive impairment in the elderly, and Alzheimer’s disease. GSH is the redox center of cells: due to the mutual transformation between the reduced state (GSH) and the oxidized state (GSSG), the redox balance can be maintained. It is also directly involved in the production of signal molecules such as H_2_S, protein functionalization, and other processes, and is a key part of intracellular gene regulation and signal transduction. An imbalance of GSH is directly associated with many diseases like cancer, cardiovascular disease, and Alzheimer’s diseases. The development of fluorescence sensors for thiols has been attracting attention in recent years due to the advantages of high sensitivity, no destructivity, in situ examination, and intracellular detection. Recently, a new luminescent sensor for the detection of Cys has been synthesized, with the introduction of methylsulfonyl and phenylsulfonyl groups onto the Cur skeleton. The detection effect was achieved through the nucleophilic attack of amino acids, and the complexation with BF_2_ group, to increase the emission wavelength of the sensors. Both probes can respond to amino acids but recognize specifically Cys, with a significant blue shift in UV max absorption and emission wavelengths and a color change visible to the naked eye [79].

**Figure 11:**
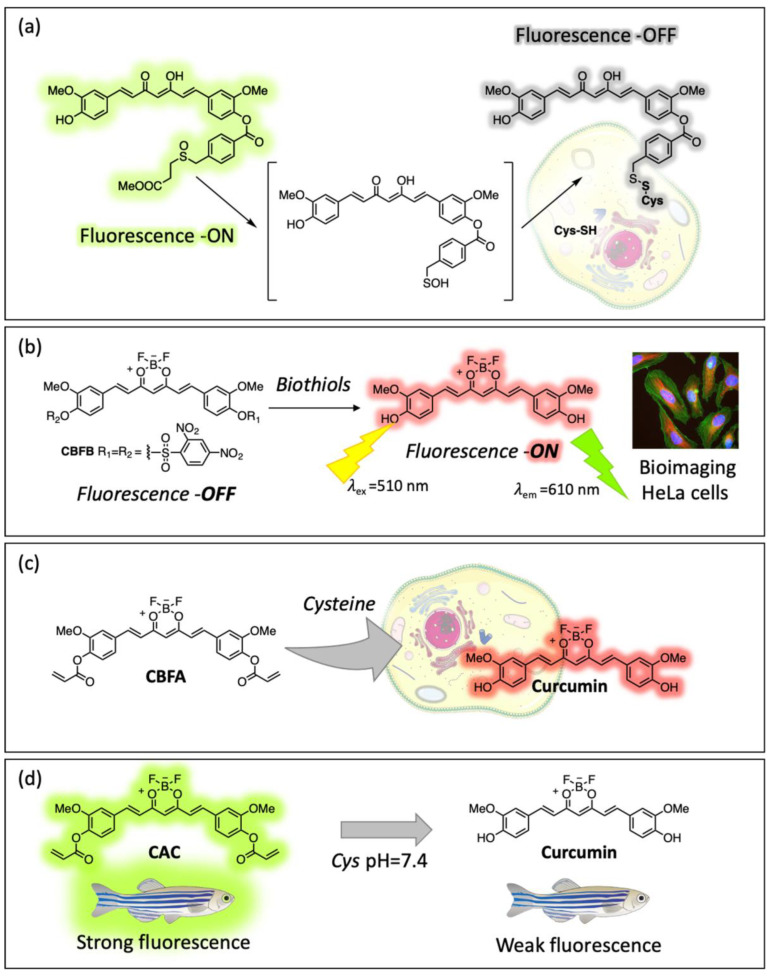
Curcumin-based sensors for biothiols. (**a**) Cur-derived sulfenic acid for Cy sensing; (**b**) 2,4-dinitrobenzene sulfonyl-Cur derivative for biothiols sensing; Cur-acrylate derivative for Cys sensing in vitro (**c**) and in vivo (**d**).

Sulfenic acids are important intermediates in synthetic [80] and biological pathways, such as oxidation processes, transcription regulation events, signal transduction processes, and catalytic and structural roles in enzymes. In the chemistry of proteins, the disulfide formation by sulfenic acids is matched by the oxidation of Cys residues. A Cur-derived sulfenic acid was generated in situ by thermolysis from suitable synthetic precursors [81] and was condensed with the thiol group of Cys and GSH by the formation of a disulfide bond. The isolation of both unsymmetrical Cur-biothyol disulfides represents a proof of concept for the feasibility of trapping transient Cur-SOH by biothiols. The quenching of the luminescence of the Cur unit in the two disulfides may also provide an on/off light switch biosensor to detect the presence of such biothiols in a cellular environment (Figure 11a) [82].

D. Chen and coworkers designed and synthesized three luminescent probes for the sensing of biothiols. The first two possess the Cur skeleton linked to the 2,4-dinitrobenzene sulfonyl moiety to one or two phenolic OH groups, for quenching the emission of the fluorophore by a photoinduced electron transfer process (Figure 11b). Theoretical calculations showed that two sulfonyl moieties are needed for a complete quenching of the luminescence, restored in the presence of biothiols because of the cleavage of the quencher group by a nucleophilic aromatic substitution reaction. The “turn on” luminescent sensor showed high sensitivity towards biothiols in living HeLa cells [83]. One year later, the same authors described the synthesis of a luminescent sensor for Cys, where the two phenolic OH groups were linked to two acrylate moieties as recognition sites for Cys, and the BF_2_ moiety was complexed to the α,β-unsaturated-diketones (Figure 11c). In this case, the Cys recognition mechanism was confirmed to be a concerted reaction such as Michael addition and intramolecular cyclization. The luminescent sensor was biocompatible, low cytotoxic, and was successfully applied to selectively imaging Cys in living MCF-7 cells [84]. A Cur-based sensor for Cys was earlier reported with a similar recognition mechanism to the last probe. The “turn off” sensor was applied to detect Cys in PC12 cells and in zebrafish, with good permeability (Figure 11d) [85].

#### 3.2.4. Curcumin-Based Sensing for Improving Human Health

Picric acid (PA) with some others aromatic nitro compounds are basic components for unexploded land mines. It is dangerous to human health but extensively used in the manufacture of pharmaceuticals, fireworks, and dyes. The detection of PA can contribute to an improvement in human life. Two Cur derivatives, specifically ferrocenyl-Cur and 4-nitro-benzylidene Cur, were synthesized as probes for PA detection. PA was shown to enhance the fluorescence intensity of the Cur-based probe through the formation of aggregates. The “turn on” 4-nitro-benzylidene Cur sensor demonstrated a good limit of detection of PA and was applied for real-time detection in water samples collected from different sources [86].

**Figure 12:**
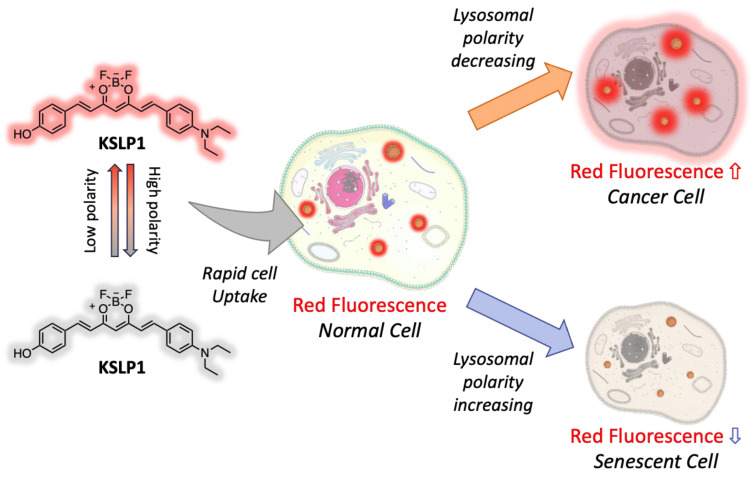
Lysosome-targeting Cur-based probe KSLP1 and its sensing mechanism.

Starting from the observation that there is a relationship between lysosomal polarity and aging, the authors of [87] designed a series of luminescent curcumin-based lysosome-targeting probes to investigate if lysosomal polarity increases in senescent cells. One of these, reported in Figure 12 and called by the authors KSLP1, appeared to be a promising near-infrared tool to monitor variations in the lysosomal polarity of MRC5 cells of *C. elegans*, providing insight into the relationship with the aging process.

A luminescent sensor for the detection of hypochloride (ClO^−^) was described. ClO^−^ is a highly reactive species that plays many roles in organisms, such as an agent in the immune system, antibacterial system, and many others in daily life, such as a disinfectant, sterilizers, or bleach. A very low ClO^−^ concentration does not guarantee bacterial protection, while an excess of ClO^−^ can be a cause of carcinogenicity or mutagenicity. The sensor was prepared by assembling carbon quantum dots and GSH, through a hydrothermal process. Cur was introduced into the NPs, achieving a decrease in the luminescence intensity of CDs (435 nm) and the appearance of an emission peak at 540 nm. The addition of ClO^−^ weakened the peak at 540 nm and restored the CD emission. The selectivity was very high for ClO^−^ and was tested in samples of pool water, tap water, and milk [88].

An excess of bisulfite ion (HSO_3_^−^), generated by the hydration of gaseous sulfur dioxide, can lead to a variety of diseases in the human body, and Cur was used as a tool for monitoring the presence and quantity of such ion. The BF_2_ complexes of Cur and demethylated Cur responded selectively to HSO_3_^−^, with a change from the blue or the pink color to colorless in aqueous buffer solution. The sensing mechanism was attributed to the addition of HSO_3_^−^ to the α,β-unsaturated-diketone moiety of the BF_2_-Cur complexes, with the formation of disulfonic acid. The bioimaging of bisulfite was performed in living H1975 cells through dual-color fluorescence imaging [89].

## 4. Conclusions and Future Directions

This review does not intend to cover all of the recent literature on the properties and applications of Cur in bioimaging and biosensing. As a matter of fact, in many fields, such as that of food, reviews have been cited that will allow the reader to delve deeper into that specific sector. The ultimate aim of the review is to maintain the interest in this natural biologically active molecule, focusing essentially on its significant photochemical characteristics. For this reason, the various synthetic routes that are reported in the literature to obtain curcuminoids from ferulic acid or vanillin derivatives are not reviewed, deliberately focusing on modifications made directly to the curcumin skeleton. Cur is a “cubistic molecule”: its nature can be dissected in all its components to represent, almost photographically, its properties from multiple angles at the same time. It has a π-conjugated structure with high electron delocalization that confers significant electrical and optical properties. In the curcumin skeleton, the reactive α,β-unsaturated-diketone moieties can be subjected to nucleophilic reactions, the phenoxy groups can be transformed into various functional groups, and each modification can deeply influence the natural luminescence of Cur. A certain space in the review, for example, has been given to the BF_2_-Cur complexes, whose peculiarities in terms of luminescence have made them, in recent years, the subject of stimulating research, especially for neurodegenerative diseases such as AD or Parkinson’s. The 1,3-diketone moiety offers the possibility of chelating Cur with various metals and oxophilic atoms, changing fluorescence emissions and intensity; this issue must be addressed in future for improving Cur probes in the fields of both bioimaging and sensing. The efforts to build bichromophoric dyes from Cur lie in the research of fluorescent small dyes that can exert considerable quantum yield, high sensitivity, chemical and photo-stability, and compatibility with living systems for future more targeted and sensitive applications. Theragnostics is a “bright spot” for this small molecule, and future concerns are certainly connected with the development of new diagnostic and healing nanomaterials. The research into biocompatible and targeting systems for imaging and drug delivery cannot be considered exhaustive, since there are still few applications in clinical trials. Most of the research has been carried out in preclinical models. More in vitro and in vivo studies are required in future before understanding the impact of curcumin-based nanoformulations on humans and their long-term risks.

The hope is to have provided the reader with a general overview of the field of application of Cur in bioimaging and sensing, highlighting what the possible future developments of research are in this regard. However, the reader must keep in mind that what are now the future directions of Cur research can quickly become the present reality, as the progress and discoveries of new applications of this molecule are so incredibly fast and engaging.
